# Introduced species will not save Caribbean coral reefs

**DOI:** 10.1073/pnas.2610820123

**Published:** 2026-05-26

**Authors:** R. Ritson-Williams, P. J. Mumby, R. S. Steneck

**Affiliations:** ^a^Department of Biology, California State University Northridge, Northridge, CA 91330; ^b^Marine Spatial Ecology Lab, School of the Environment, The University of Queensland, St. Lucia, QLD 4072, Australia; ^c^Darling Marine Center, University of Maine, Walpole, ME 04573

The opinion article by Camacho et al. (1) suggests that Pacific species of corals should be moved to the Caribbean to “fix” the coral reef crisis. Until the Caribbean species are ecologically extinct across their entire range, introducing novel species into the Caribbean could likely exacerbate the problem and cause unintended consequences. Instead, we should focus on restoring select native species and their habitat.

Shallow tropical reef-building corals have experienced a drastic decline due to the combined effects of climate change, habitat degradation, sedimentation, overfishing, and disease. Fundamentally, addressing climate change should be our first priority for the long-term persistence of corals, but it involves a massive shift in how human societies operate; “an inconvenient truth” is an understatement. Local conditions can magnify the impacts of climate change on coral survival (2). Even a transplanted coral will not survive in a hostile habitat. Restoring Caribbean corals is a complex problem that deserves a multifaceted response: scaling up of adaptive restoration (3), genetic rescue (4), and selective breeding (5) likely all have a role in increasing the genetic diversity of native species.

**Fig. 1.**
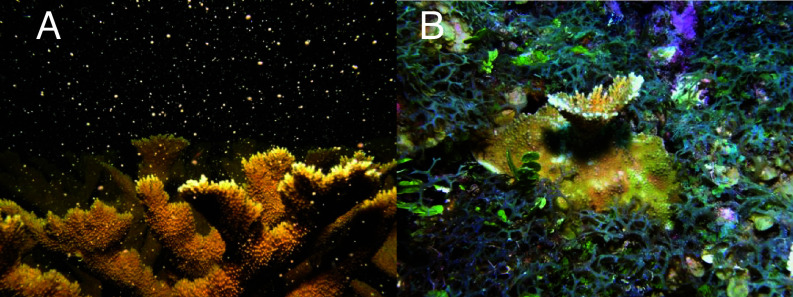
Not Dead Yet! (*A*) In Belize there are healthy stands (although smaller than historical abundance) of the framework building *A. palmata* that spawn almost every summer. (*B*) Many Caribbean corals are competing with fast growing algae, which could pose a major competitive obstacle for an introduced Pacific coral species, just as it has for the native species.

Coral reef ecosystems need natural ecological processes to function (6). Simply planting corals is not enough, we need to restore herbivory and multiple trophic levels, minimize competition and maximize facilitation, and improve adult density to promote successful coral spawning and recruitment in situ. Planting Indo-Pacific corals on a Caribbean reef might improve habitat complexity but also could create competitors for the surviving corals. Camacho et al. argue we are in the 11th hour for Caribbean reefs, but we continue to see *Acropora spp.* in the tropical Caribbean (Fig. 1), despite being effectively extinct from the subtropical reefs of Florida (7). If Caribbean coral species no longer provide ecosystem services across their entire range, perhaps an introduced species could be a restoration tool, but introducing foreign species now is unproven and could create unintended consequences.

Transplanting organisms across geographic barriers has a long history of failure (8). Camacho et al. argue that we should start with controlled experiments in mesocosms. Absolutely, this precautionary approach is critical but the broader challenge lies in being comprehensive. Mesocosm experiments will not be able to simulate the range of potential unintended consequences. For example, would introduced species impact nonscleractinian habitat-forming taxa including octocorals, sponges, and zoanthids? History shows that the Caribbean is an immunosuppressed region, with devastating epizootics wiping out diademid urchins in the Atlantic but not Pacific (9), and widespread coral diseases in the region (10). Should we risk the extant coral populations of other Caribbean nations by introducing a “superrecruiter” species to Florida? In marine systems, there are few barriers to dispersal (for instance the rapid spread of introduced lionfish from Florida across the Caribbean). We would warn against rash actions that contribute to the very ecosystem collapse that we are working to remedy. Instead, we need a sharper focus on fixing the habitat and maintaining both genetic diversity and ecological processes to avoid the extinction of native coral species.
